# Worsening symptoms in ADHD children caused by increased parental stress before, during and after Covid-19

**DOI:** 10.1192/j.eurpsy.2024.947

**Published:** 2024-08-27

**Authors:** R. Tarazhi

**Affiliations:** Child and Adolescent Psychiatry, Mother Theresa University Hospital, Tirana, Albania

## Abstract

**Introduction:**

Attention Deficit Hyperactivity Disorder (ADHD) is a neurodevelopmental disorder characterized by high levels of inattention, hyperactivity, and impulsivity that are present before the age of seven, seen in a variety of situations, inconsistent with the child’s developmental level and that cause social or academic damage. Parents may respond with high levels of verbal aggression and disciplinary measures to disruptive behaviors, which causes their children to respond negatively, influencing a bidirectional process of participating of a vicious circle. The pandemic has been a huge battle for everyone. Their anxiety in this extraordinary situation can also increase the children’s psychological and behavioral problems.

**Objectives:**

This literature review aims to explore the connection between the increase of parental stress among parents of ADHD children and worsening symptoms of ADHD, before and during COVID-19 outbreaks.

**Methods:**

The literature review was performed by searching the following electronic databases (for all available years from 2005-2021): PubMed, PubMed Central, Springer Open, Hindawi, Google Scholar. We included studies with a primary focus on parenting stress in families that have children, aged 6-12 years old, with a clinical diagnosis of ADHD that was made by a specialist using the diagnostic criteria of DSM-III/DSM-IV/DSM-V or ICD-10. The search was organized in chronological order by selecting studies published in the time period before,during and after the pandemic.

**Results:**

Parents of children with ADHD tend to use inappropriate parenting styles, they are more disapproving, critical and exhibit poorer monitoring and more corporal punishment than parents of children without ADHD who try to control disruptive behaviors. These parenting styles can affect the course of the disease, worsen its manifestations and cause the secondary development of psychiatric and maladaptive behaviors. In some of the studies,during the outbreak of the COVID-19 pandemic, is observed a high prevalence of depressive symptoms (62.5%) among caregivers, while 20.5% and 36.4% indicated anxiety and stress symptoms, respectively.Some parents reported deterioration of general well-being in their children and this manifested as oppositional/defiant attitudes and emotional outbursts, sleep problems and anxiety in this context.

**Conclusions:**

The pandemic has had psychological influences on parents with ADHD that affected their children’s compliance with the medication and, consequently worsened their symptomatology. Society can be exposed to chronic stressors like Covid-19 anytime soon, so the main focus must be  identifying needs to inform future interventions designed to support parents and ultimately their children. Psychoeducation of parents should be promoted in order to cope with the symptomatology of ADHD in the field of normality or under a chronic stressor.

**Disclosure of Interest:**

None Declared

